# Transcription Coactivator ANGUSTIFOLIA3 (AN3) Regulates Leafy Head Formation in Chinese Cabbage

**DOI:** 10.3389/fpls.2019.00520

**Published:** 2019-04-30

**Authors:** Jing Yu, Liwei Gao, Wusheng Liu, Lixiao Song, Dong Xiao, Tongkun Liu, Xilin Hou, Changwei Zhang

**Affiliations:** ^1^State Key Laboratory of Crop Genetics and Germplasm Enhancement, College of Horticulture, Nanjing Agricultural University, Nanjing, China; ^2^Department of Horticultural Science, North Carolina State University, Raleigh, NC, United States; ^3^Jiangsu Key Laboratory for Horticultural Crop Genetic Improvement, Nanjing, China

**Keywords:** *AN3*, *BRM*, leafy head formation, VIGS, transcriptome analysis, Chinese cabbage

## Abstract

Leafy head formation in Chinese cabbage (*B. rapa* ssp. *pekinensis* cv. Bre) results from leaf curvature, which is under the tight control of genes involved in the adaxial-abaxial patterning during leaf development. The transcriptional coactivator ANGUSTIFOLIA3 (AN3) binds to the SWI/SNF chromatin remodeling complexes formed around ATPases such as BRAHMA (BRM) in order to regulate transcription in various aspects of leaf development such as cell proliferation, leaf primordia expansion, and leaf adaxial/abaxial patterning in Arabidopsis. However, its regulatory function in Chinese cabbage remains poorly understood. Here, we analyzed the expression patterns of the Chinese cabbage *AN3* gene (*BrAN3*) before and after leafy head formation, and produced *BrAN3* gene silencing plants by using the turnip yellow mosaic virus (TYMV)-derived vector in order to explore its potential function in leafy head formation in Chinese cabbage. We found that *BrAN3* had distinct expression patterns in the leaves of Chinese cabbage at the rosette and heading stages. We also found silencing of *BrAN3* stimulated leafy head formation at the early stage. Transcriptome analysis indicated that silencing of *BrAN3* modulated the hormone signaling pathways of auxin, ethylene, GA, JA, ABA, BR, CK, and SA in Chinese cabbage. Our study offers unique insights into the function of *BrAN3* in leafy head formation in Chinese cabbage.

## Introduction

Heading Chinese cabbage (*Brassica rapa* ssp. *pekinensis* cv. Bre; 2n = 2× = 20) is one of the most important horticultural crops in China and, to a lesser extent, an oilseed crop (Zhao et al., 2005). Leafy head formation undergoes four developmental stages, i.e., the seedling, rosette, folding, and heading stages (He et al., 2000; Yu et al., 2000; Ke, 2010; Wang et al., 2014b). It forms uniform leafy heads with extremely incurved leaves surrounding the shoot apexes after the rosette stage. The leafy heading trait has been selected for several *Brassica* species including Chinese cabbage and cabbage (*B. oleracea*; 2n = 2× = 18) during crop domestication and breeding (Cheng et al., 2014). Well-formed leafy heads function normally for photosynthesis and serve as storage organs for essential minimal nutrients, fibers, and vitamins, while poorly-developed heads significantly decrease the crop’s commercial values (Yu et al., 2000, 2013; Cheng et al., 2016). Leafy head formation is affected by many factors such as low temperature, low light intensity, uneven auxin distribution, and low carbohydrate/nitrogen ratio (Ito, 1957). Plants have to respond to these factors properly for leaf bending and folding (i.e., leaf curvature), which results from the differential cell division and enlargement in leaf regions, and which are under the tight control of genes involved in the adaxial-abaxial patterning during leaf development (Mao et al., 2014).

Recent efforts had been taken to study the molecular mechanisms controlling leafy head formation in Chinese cabbage by using genetic mapping (Zhao et al., 2005; Ge et al., 2011; Yu et al., 2013; Zhang et al., 2013; Zhang X. et al., 2016), transcriptome profiling (Wang et al., 2012; Zhang C.W. et al., 2016; Gu et al., 2017; Li et al., 2018), proteomic analysis (Zhang C.W. et al., 2016), and miRNA expression profiling (Wang et al., 2013). Moreover, the effects of a few individual genes on leafy head formation had been studied in Chinese cabbage. These genes include the *Agrobacterium* genes *Aux1* and *Aux2* (He et al., 2000), the Chinese cabbage genes *BcpLH* (Yu et al., 2000), *TCP* (Mao et al., 2014), *BrpSPL9* (Wang et al., 2014b), and auxin transport genes *BrLAX, BrPIN*, and *BrPGP* (Gao et al., 2017). Most of these genes are involved in the adaxial-abaxial patterning during leaf development in Chinese cabbage.

The Arabidopsis *ANGUSTIFOLIA3/GRF-INTERACTIN FACTOR1* (*AtAN3*/*AtGIF1*), an important transcriptional activator of the GIF family, is also involved in the determination of leaf adaxial-abaxial patterning and growth (Horiguchi et al., 2011). Loss-of-function mutant of *AtAN3* exhibited smaller and narrower leaves due to a decrease in cell number (Kim and Kende, 2004; Horiguchi et al., 2005), while ectopic overexpression of *AtAN3* resulted in enlarged leaf size (Horiguchi et al., 2005; Lee et al., 2009). AtAN3 binds to the SWI/SNF chromatin remodeling complex formed around ATPases such as BRAHMA (BRM). By using the energy from ATP hydrolysis, the Arabidopsis AN3-SWI/SNF-BRM complex regulates gene expression by changing the interactions between histone octamers and the DNA for the access of transcription factors (Clapier and Cairns, 2009). The *brm* mutant had small spiral-shaped leaves with downward curling edges (Hurtado et al., 2006; Vercruyssen et al., 2014). The AN3-SWI/SNF-BRM complex also interacts with GROWTH REGULATING FACTOR (GRF) proteins, a class of plant-specific transcription activators in Arabidopsis (Kim and Kende, 2004; Liu et al., 2009; Debernardi et al., 2014). Ectopic overexpression of the GRFs increased leaf size in Arabidopsis due to enhanced cell proliferation (Kim et al., 2003; Horiguchi et al., 2005; Liu et al., 2009; Debernardi et al., 2014; Wang et al., 2014a). However, the regulatory function of *AN3* in Chinese cabbage remains poorly understood.

In the present study, we explored the expression patterns of the Chinese cabbage *AN3* gene (i.e., *BrAN3*) in different leaf locations of Chinese cabbage at the rosette and heading stages. We generated *BrAN3-*silencing Chinese cabbage plants by using the turnip yellow mosaic virus (TYMV)-derived vector, and examined the effect of *BrAN3* silencing on the stimulation of leafy head formation. We also conducted transcriptome analysis of the *BrAN3-*silencing leaves and identified the differentially expressed genes (DEGs) caused by *BrAN3* silencing. All of the results provide insights into the function of the *BrAN3* gene in leafy head formation in Chinese cabbage.

## Materials and Methods

### Sequence Alignment and Phylogenetic Analysis

The protein sequences of the Arabidopsis *AtAN3* and *AtBRM* genes were used individually as the query sequences to BlastP against the *Brassica* database^1^ in order to obtain their homologous sequences in *B. rapa*. The protein sequences of the Arabidopsis *AtAN3* and *AtBRM* genes were also used individually as the query sequences to BlastP against Phytozome^2^ in order to obtain their homologous sequences in *B. oleracea*, *Zea mays*, and *Oryza sativa*. The genomic DNA sequences and cDNA sequences of these homologous sequences in these plant species were also obtained from Phytozome. All of the homologous protein sequences of each gene in different species were aligned together using CluastalX 2.1 (Larkin et al., 2007), and subjected to the phylogentic analysis using the MEGA5 program^3^ and the neighbor-joining method with 1,000 bootstraps (Tamura et al., 2013).

### Plant Material and Growth Conditions

Inbred line seeds of Chinese cabbage cv. Bre were germinated in soil in a tray with holes. Three weeks old seedlings were transferred to pots containing a mixture of nutrient soil and vermiculite (3:1) and grown in a growth chamber at 22°C with a 16/8 h light/dark photocycle and 50% relative humidity.

### Gene Cloning and Plasmid Construction

Total RNA was extracted from the leaves of Chinese cabbage for gene cloning using an RNA extraction kit (TaKaRa; Dalian, Liaoning, China). DNase I treatment and first-strand cDNA synthesis were performed sequentially using the PrimeScript^®^ 1st Strand cDNA Synthesis Kit (TaKaRa; Dalian, Liaoning, China). One microgram of total RNA was reverse-transcribed with the oligo(dT) primer according to the manufacturer’s protocol. The cDNA fragments of *BrAN3* and *BrBRM* were PCR amplified individually using the first-strand cDNA as the templates and the gene-specific primers (Supplementary Table S1). The PCR products were purified using an AxyPrep DNA Gel Extraction Kit (Axygen Biosciences; Union City, CA, United States) and then cloned into the pMD19-T vector (TaKaRa; Dalian, Liaoning, China), followed by Sanger sequencing. A gene-specific fragment of 40 nt in length was selected for each of the two genes to create an in-frame stop codon (TAA, TGA, or TAG) on the second, third or fourth amino acid position on each fragment due to frame shift. The gene-specific fragment was selected to target all the homologous sequences of each gene in the Chinese cabbage genome. Each fragment and its reverse complementary sequence (Supplementary Table S1) were synthesized from TaKaRa (Dalian, Liaoning, China) and used to form a palindromic oligonucleotide dimer after self-hybridization, which was cloned into the plasmid pTY-S with the help of *Sna*BI (Pflieger et al., 2008). The resulting plasmids were named as pTY-*BrAN3* and pTY-*BrBRM*, respectively.

### Real-Time RT-PCR

Total RNA was extracted from the apical location, the lateral 1∼3 locations, and the base location of individual leaves and the whole leaves of Chinese cabbage (Gao et al., 2017) at both the rosette and heading stages, respectively, for the analysis of expression profiles of *BrAN3* on different locations of Chinese cabbage leaves. As for the analysis of the effects of virus-induced gene silencing, total RNA was extracted individually from the Chinese cabbage leaves inoculated with each virus-induced gene silencing vector, the negative control vector, and positive control vector. The purity and integrity of the RNA were analyzed using a NanoDrop ND-1000 spectrophotometer (NanoDrop Technologies; Wilmington, DE, United States) and verified by gel electrophoresis. The real-time RT-PCR was conducted using the SYBR Green PrimeScript Plus RT-PCR Kit (TaKaRa; Dalian, Liaoning, China) on an ABI PrismR 7900HT (Applied Biosystems; Carlsbad, CA, United States) according to the manufacturer’s instructions. Gene-specific primers were designed outside of the 40 nt long fragments for both *BrAN3* and *BrBRM* genes as well as the *BrAN3* downstream genes *ARABIDOPSIS RESPONSE REGULATOR4* (*ARR4*) and *CYTOKININ RESPONSE FACTOR2* (*CRF2*) (Vercruyssen et al., 2014) using the Beacon Designer 7.9 (Premier Biosoft International, Palo Alto, CA, United States) (Supplementary Table S1). These primers were designed to target all the homologous sequences of each gene in the Chinese cabbage genome. The amplification procedure was as follows: pre-denaturation at 94°C for 10 s, followed by 40 cycles of 94°C for 30 s and 60°C for 30 s, and finally a melting curve was performed (95°C for 15 s and at increments of 0.5°C from 60 to 95°C). Three biological and three technical replicates were carried out for each sample. The 2^-ΔΔCT^ method (Schmittgen and Livak, 2008) was used for relative transcript quantification normalized by the internal control gene *BrAct* (*Bra028615*) as described (Dheda et al., 2004). The quality cutoff of the real-time RT-PCR efficiency was set at >95% with *R*^2^ > 0.99 for the standard curve.

### Particle Bombardment-Mediated Delivery of the TYMV-Derived Vectors

The plasmids pTY-*BrAN3* and pTY-*BrBRM*, the empty plasmid pTY-S and the positive control plasmid pTY-*BrPDS* (Yu et al., 2018) were transformed into the 6 weeks old Chinese cabbage seedlings for virus-induced gene silencing (VIGS) by particle bombardment as described in Yu et al. (2018). Leaves were harvested from three individual plants before and after head formation and kept in liquid nitrogen for RNA extraction (see above).

### Library Preparation for Transcriptome Sequencing

Total RNA was extracted individually from the leaves of two Chinese cabbage plants inoculated with each virus-induced gene silencing vector. The purity and integrity of the RNA was assessed using the RNA Nano 6000 Assay Kit of the Agilent Bioanalyzer 2100 system (Agilent Technologies; Palo Alto, CA, United States) and the NanoPhotometer^®^ spectrophotometer (IMPLEN; Westlake Village, CA, United States), while the concentration was measured using the Qubit^®^ RNA Assay Kit in a Qubit^®^ 2.0 Flurometer (Life Technologies; Carlsbad, CA, United States). Three μg RNA per sample was used for RNA sample preparations. Sequencing libraries were generated using the NEB Next^®^ Ultra^TM^ RNA Library Prep Kit for Illumina^®^ (NEB; Beverley, MA, United States) following the manufacturer’s recommendations and index codes were added to attribute sequences to each sample. The library quality was assessed on the Agilent Bioanalyzer 2100 system (Agilent Technologies; Palo Alto, CA, United States).

### Clustering and Reads Alignment to the Reference Genome

The clustering of the index-coded samples was performed on a cBot Cluster Generation System using TruSeq PE Cluster Kit v3-cBot-HS (Illumina; Hayward, CA, United States) according to the manufacturer’s instructions. Then, the libraries were sequenced on an Illumina Hiseq 2500 platform and 150 bp paired-end reads were generated. Reference genome and gene model annotation files were downloaded from *Brassica* database^4^. Index of the reference genome^4^ was built and paired-end clean reads were aligned to the reference genome using HISAT2 (Kim et al., 2015).

The expression level was calculated as fragments per kilobase of exon model per million mapped (FPKM) values and gene expression pattern using Clusterv3.0^5^ and the heat map of hierarchical clustering was established using TreeView v.3.0^6^ based on the log2-converted FPKM values. Bubble Charts were performed using the OmicShare tools, a free online platform for data analysis^7^.

### GO Enrichment and KEGG Pathway Analysis of Differentially Expressed Genes

Differentially expressed genes (DEGs) were defined if the False Discovery Rate is smaller than 0.01 and the Fold Change equals to or is larger than 2. Gene Ontology (GO) enrichment analysis of the DEGs was implemented by the GOseq R packages based on the Wallenius non-central hyper-geometric distribution (Young et al., 2010), which allows the adjustment for gene length bias in DEGs. KOBAS software was used to test the statistical enrichment of DEGs in KEGG pathways (Mao et al., 2005).

### Statistical Analysis

Statistical analyses was performed using analysis of variance (ANOVA; SAS 9.4 for Windows; SAS Institute, Cary, NC; *p*<0.05).

## Results

### Identification of the *BrAN3* and *BrBRM* Genes in Chinese Cabbage

**FIGURE 1:**
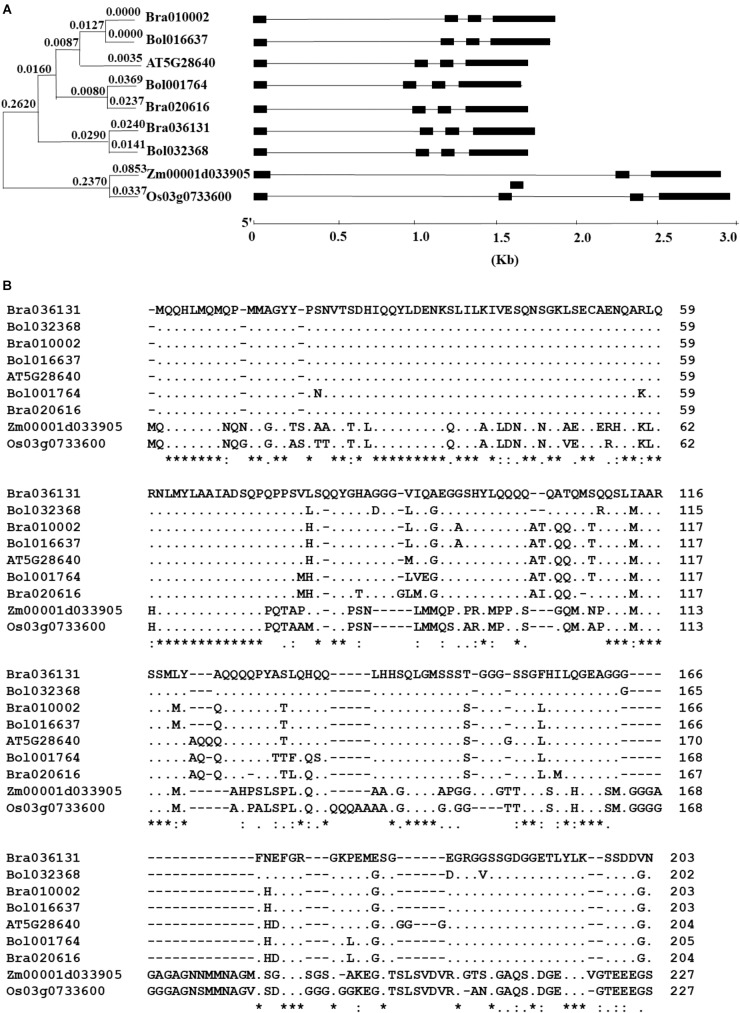
Comparison of the homologs of the AtAN3 protein in *Brassica rapa*, *B. oleracea*, *Zea mays*, and *Oryza sativa*. **(A)** Phylogenetic relationship and gene structures of the homologs of the AtAN3 protein in those species. The unrooted phylogenetic tree was generated by the neighbor-joining method using the MEGA5 program with 1,000 bootstraps. Lines represent the introns while solid boxes represent exons. **(B)** Comparison of the deduced amino acid sequences of the homologs of the AtAN3 protein in those species. Dashes represent gaps, dots represent the identical amino acid residues, and the numbers represent the length of the amino acid residues.

The BlastP search for the homologous sequences of the *AtAN3* gene in the *Brassica* database and Phytozome returned three homologous genes in both *B. rapa* and *B. oleracea*, and one homologous gene in both *Z. mays* and *O. sativa* (Figure 1). Phylogenetic analysis revealed that the homologous genes in *B. rapa* and *B. oleracea* formed three monophyletic groups, indicating an origin of gene duplication (Figure 1A). The solo Arabidopsis homolog grouped with one of the three groups. The comparison between the cDNA sequences with the genomic DNA sequences revealed that all of these homologous genes have similar exon/intron structures (Figure 1B). Each homologous gene consists of four exons and three introns at relatively conserved positions. When using the protein sequence of the *AtBRM* gene as the query, we found two homologs in both *B. rapa* and *B. oleracea*, and one homolog in both *Z. mays* and *O. sativa* (Supplementary Figure S1). The homologous genes in *B. rapa* and *B. oleracea* formed two monophyletic groups, indicating a single gene duplication event (Supplementary Figure S1). All of these homologous genes have similar exon/intron structures (Supplementary Figure S1). The existence of the same numbers of homologous genes of both *AtAN3* and *AtBRM* in the two Brassica genomes is consistent to the finding that parallel selection of homologous genes for leaf heading in the two species (Cheng et al., 2016).

### Distinct Expression Patterns of *BrAN3* in the Leaves of Chinese Cabbage at the Rosette and Heading Stages

**FIGURE 2:**
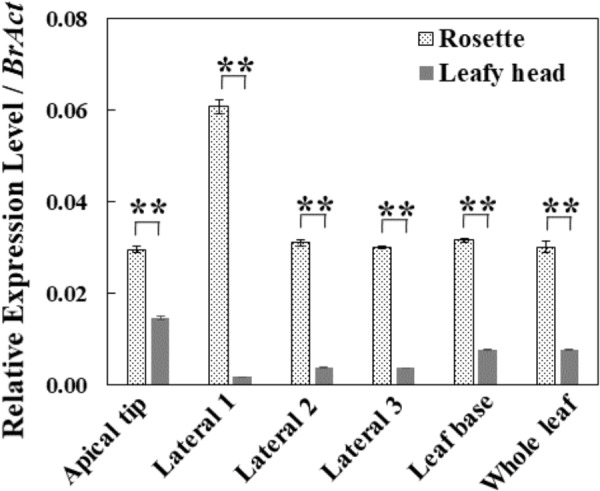
Relative expression levels of *BrAN3* in different leaf positions and the whole leaf of Chinese cabbage at the rosette and heading stages measured by real-time RT-PCR. Total RNA was extracted from the apical location, the lateral 1∼3 locations, and the base location of individual leaves and the whole leaves of Chinese cabbage (Gao et al., 2017) at both the rosette and heading stages, respectively. *BrAN3*-specific primers were designed to target all the three homologous sequences of *BrAN3* in Chinese cabbage. Relative expression levels were calculated using the standard curve method with *BrAct* (*Bra028615*; Dheda et al., 2004) as the internal control gene. Bars represent the means of three replicates ± standard errors (vertical bars). Asterisks indicate a significant difference between the two stages at *p* ≤ 0.05 as calculated by *t*-test.

To explore the potential roles of the *BrAN3* gene in head formation in Chinese cabbage, we conducted real-time RT-PCR analysis of the relative transcription levels of *BrAN3* in the Chinese cabbage leaves at both the rosette and heading stages. We found that *BrAN3* had a constantly stable relative expression levels in different locations of the leaves as well as the whole leaf at the rosette stage except in the leaf apical tip where *BrAN3* expression levels doubled (Figure 2). However, *BrAN3* exhibited highly variable but significantly decreased relative expression levels in different locations of the leaves of Chinese cabbage at the heading stage (Figure 2). Their relative expression peaked in the leaf apical tip, followed by the leaf base, and the lateral 2 and 3 locations. The least relative expression was observed in the lateral 1 location. The significant variations on the relative expression in different locations of the Chinese cabbage leaf at the heading stage indicate a potential role in heading formation since the lateral parts affect the leaf curvature and leaf head formation than the mid-vein part (i.e., the lateral 1 location) does.

### The Effects of VIGS-Mediated Knockdown of *BrAN3* and *BrBRM* Individually in Head Formation in Chinese Cabbage

**FIGURE 3:**
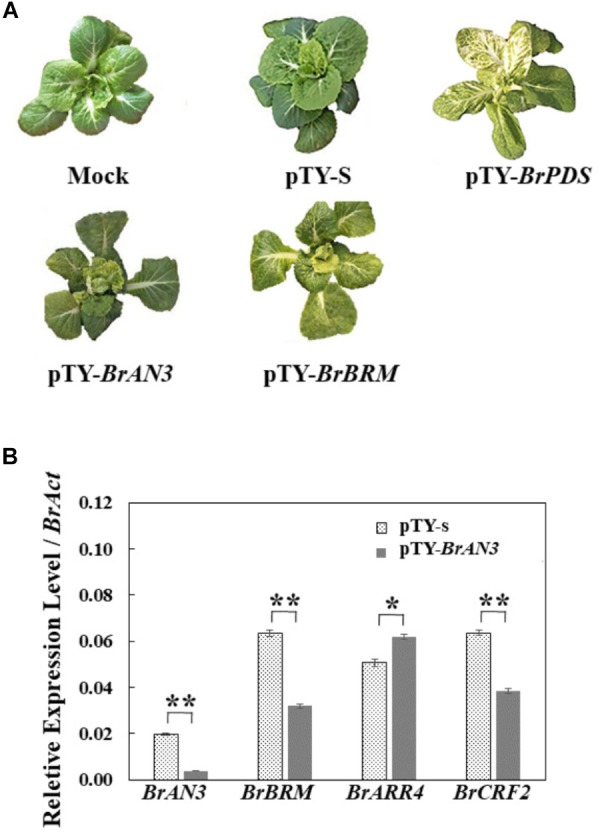
Effects of gene silencing of *BrAN3* and *BrBRM* in Chinese cabbage. **(A)** Representative images of the silencing plants by using the TYMV-derived vector pTY-S at 60 DPI. A gene-specific fragment of 40 nt in length was selected for each of the two genes to create an in-frame stop codon (TAA, TGA, or TAG) on the second, third or fourth amino acid position on each fragment due to frame shift. The gene-specific fragment was selected to target all the homologous sequences of each gene in the Chinese cabbage genome. Six weeks old Chinese cabbage plants were inoculated with each gene silencing vector. The mock treatment and the empty vector pTY-S were used as the negative control, while the pTY-*BrPDS* was used as the positive control. Leaf head formation was observed for pTY-*BrAN3* and pTY-*BrBRM*-inoculated plants. **(B)** Relative expression levels of *BrAN3*, *BrBRM*, and two of *BrAN3* downstream genes *BrARR4* and *BrCRF2* in the *BrAN3*-silencing plants measured by Real-time RT-PCR. RNA was extracted from the *BrAN3*-silencing leaves with the empty vector pTY-S-inoculated leaves as the control at 60 DPI. Relative expression levels were calculated using the standard curve method with *BrAct* (*Bra028615*; Dheda et al., 2004) as the internal control gene. Bars represent the means of three replicates ± standard errors (vertical bars). Asterisks indicate a significant difference between the two stages at *p* ≤ 0.05 as calculated by *t*-test.

To further explore the potential roles of the *BrAN3* and its genetically interacting *BrBRM* gene in head formation in Chinese cabbage, VIGS was employed to knock down the expression of *BrAN3* and *BrBRM* individually in Chinese cabbage. At 15 days post inoculation (DPI), all the TYMV-derived vector-inoculated plants displayed typical viral symptoms when compared with the untreated plants. These include plant stunting, leaf distortion, and mosaic symptoms. At 60 DPI, the untreated and the empty vector pTY-S-inoculated plants showed a normal increase in leaf size, while the positive control vector pTY-*BrPDS*-inoculated plants exhibited a photobleaching phenotype as expected (Figure 3A; Yu et al., 2018). However, the pTY-*BrAN3*- and pTY-*BrBRM*-inoculated plants produced curled leaves and formed smaller leafy heads at the rosette age (Figure 3A), indicating an important role for both genes in head formation at the early stage.

To confirm the VIGS effect of *BrAN3* in the *BrAN3*-silencing Chinese cabbage, real-time RT-PCR was performed to detect the relative expression levels of *BrAN3* in pTY-*BrAN3*-inoculated plants, respectively. When compared to its relative expression levels on the empty vector pTY-S-inoculated plants, the relative expression levels of *BrAN3* were significantly decreased on the pTY-*BrAN3-*inoculated plants (Figure 3B). In addition, we found that the relative expression levels of *BrBRM* significantly decreased in the *BrAN3*-silencing plants (Figure 3B), indicating *BrAN3* positively regulates the expression of *BrBRM*.

### The Involvement of the Cytokinin Signaling Pathway in Head Formation in Chinese Cabbage

Since *AtAN3* plays an important role in the regulation of cytokinin signaling to stimulate leaf cell proliferation and adaxial-abaxial patterning, it represses and induces the expression of its downstream genes *AtARR4* and *AtCRF2*, respectively, which are cytokinin response regulators (Rashotte et al., 2006; Werner and Schmuelling, 2009; Holst et al., 2011; Vercruyssen et al., 2014). Thus, real-time RT-PCR was also conducted to detect the relative expression levels of *BrARR4* and *BrCRF2* in the *BrAN3*-silencing plants. As expected, the relative expression levels of *BrARR4* and *BrCRF2* significantly increased and decreased, respectively, in the *BrAN3-*silencing plants when compared to the empty vector pTY-S-inoculated plants (Figure 3B).

### RNA-Seq Analysis and Differentially Expressed Gene (DEG) Identification in the *BrAN3-*Silencing Chinese Cabbage

**FIGURE 4:**
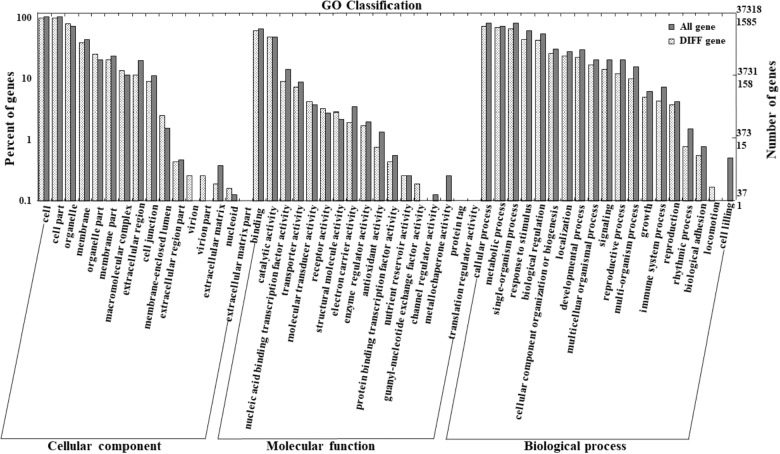
Functional Classification of the DEGs by Gene Ontology (GO) Enrichment Analysis. RNA was extracted from the *BrAN3*-silencing leaves with the empty vector pTY-S-inoculated leaves as the control at 60 days post inoculation (DPI), and used for library construction and RNA-Seq analysis. GO enrichment analysis of the DEGs was implemented by the GOseq R packages based on the Wallenius non-central hyper-geometric distribution (Young et al., 2010), which allows the adjustment for gene length bias in DEGs. It assigned all the genes and the DEGs to the three GO categories: biological processes, cell components, and molecular functions.

To further characterize the molecular mechanisms of *BrAN3* in leafy head formation, RNA-Seq was performed on the *BrAN3-*silencing plants with the empty vector pTY-S-inoculated plants being used as the control. About 20,992,641 and 19,553,742 clean reads obtained from the *BrAN3-*silencing plants and the control plants, respectively, were mapped against the Brassica reference sequence, indicating equal match ratios on the reference genome for both groups of data. When compared to the control plants, a total of 1692 DEGs were detected in the *BrAN3-*silencing plants based on the transcript abundances; among which, 800 DEGs were significantly up-regulated and 892 DEGs were significantly down-regulated (Figure 4).

### Functional Classification of DEGs by Gene Ontology (GO) Enrichment Analysis and KEGG Pathway Analysis

**FIGURE 5:**
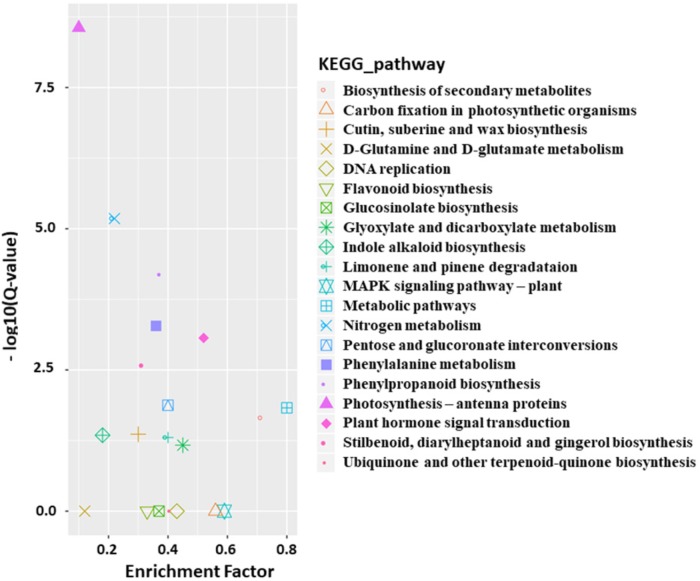
Over-represented KEGG terms of the differentially expressed genes (DEGs). KOBAS software was used to test the statistical enrichment of DEGs in KEGG pathways (Mao et al., 2005).

**FIGURE 6:**
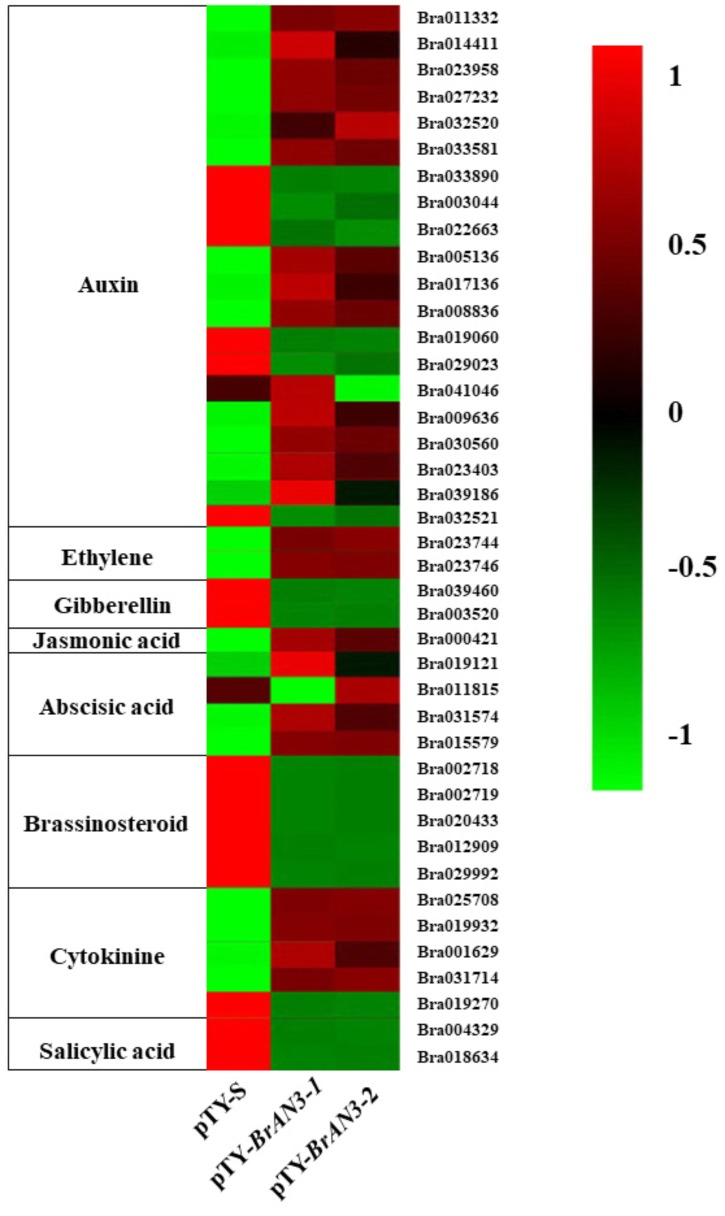
Heatmap showing the differential expression patterns of the 41 differentially expressed genes (DEGs).

Functional classification of the identified DEGs using the GO term enrichment analysis assigned all the genes and the DEGs to the three GO categories: biological processes, cell components, and molecular functions (Figure 4). Overall, DEGs was more enriched in biological processes category than that in the other two categories as well as that in all genes background. Specifically, the over-represented (*p* < 0.05) GO terms of biological processes category included cellular process, metabolic process, single-organism process, response to stimulus, biological regulation, developmental process, etc., indicating the silencing of *BrAN3* may affect the developmental and biological processes. Furthermore, the KEGG pathway analysis of the identified DEGs showed that the DEGs involving in the pathways of photosynthesis-antenna proteins, nitrogen metabolism, phenylpropanoid biosynthesis, phenylalanine metabolism, and plant hormone signal transduction were significantly enriched in the *BrAN3*-silencing plants (Figure 5). We found there existed 41 DEGs in the plant hormone signal transduction term with 23 and 16 DEGs being significantly up-regulated and down-regulated, respectively (Figure 6). The remaining 2 DEGs showed controversial results, i.e., a significant increase in one *BrAN3*-silencing plant and a significant decrease in the other *BrAN3*-silencing plant (Figure 6). Specifically, the DEGs involved in the signaling pathways of ethylene (ETH; *Bra023744* and *Bra023746*) and jasmonic acid (JA; *Bra000421*) were significantly increased in the *BrAN3*-silencing leaves when compared to the empty vector pTY-S-inovulated leaves (Figure 6). The DEGs involved in the signaling pathways of gibberellin (GA; *Bra039460* and *Bra003520*), brassinosteroid (BR; *Bra002718*, *Bra002719*, *Bra020433*, *Bra012909*, and *Bra029992*), and salicylic acid (SA; *Bra004329* and *Bra018634*) were significantly decreased in *BrAN3*-silencing leaves in comparison to the empty vector-inoculated leaves (Figure 6). However, the DEGs involved in the signaling pathways of auxin (AUX), abscisic acid (ABA), and cytokinin (CK) showed opposite expression patterns. For example, 13 DEGs involved in the AUX signaling pathway (*Bra011332*, *Bra014411*, *Bra023958*, *Bra027232*, *Bra032520*, *Bra033581*, *Bra005136*, *Bra017136*, *Bra008836*, *Bra009636*, *Bra030560*, *Bra023403*, and *Bra039186*), 3 DEGs involved in the ABA signaling pathway (*Bra019121*, *Bra031574*, and *Bra015579*), and 4 DEGs involved in the CK signaling pathway (*Bra025708*, *Bra019932*, *Bra001629*, and *Bra031714*) were significantly increased in the *BrAN3*-silencing leaves (Figure 6). And 6 and 1 DEGs in the signaling pathways of AUX (*Bra033890*, *Bra003044*, *Bra022663*, *Bra019060*, *Bra029023*, and *Bra032521*) and CK [*Bra019270* (*BrCRF2*)] showed a significantly decrease in the *BrAN3*-silencing plants (Figure 6). In addition, 1 DEG involved in each of the auxin (*Bra041046*) and ABA (*Bra011815*) signaling pathways exhibited controversial expression patterns when compared to the empty vector-inoculated leaves (Figure 6).

**FIGURE 7:**
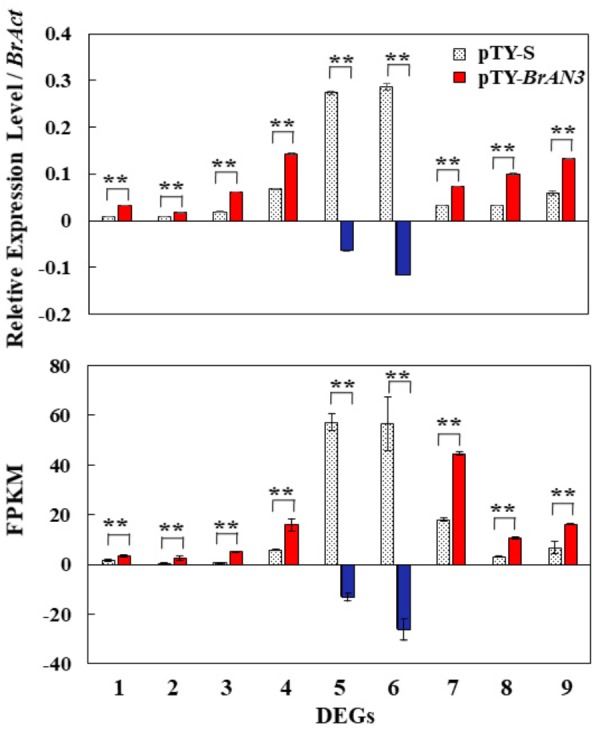
Validation of the expression of 9 out of the 41 differential expression genes (DEGs) by real-time RT-PCR. Gene-specific primers were designed to target all the homologous sequences of each gene in Chinese cabbage. Relative expression levels were calculated using the standard curve method with *BrAct* (*Bra028615*; Dheda et al., 2004) as the internal control gene. The FPKM expression levels were calculated as fragments per kilobase of exon model per million mapped (FPKM) values. Bars represent the means of three replicates ± standard errors (vertical bars). Genes: 1, *Bra011332*; 2, *Bra014411*; 3, *Bra023958*; 4, *Bra027232*; 5, *Bra003044*; 6, *Bra022663*; 7, *Bra005136*; 8, *Bra017136*; 9, *Bra025708*. Asterisks indicate a significant difference between pTY-S- and pTY-*BrAN3*-inoculated plants at *p* ≤ 0.01 as calculated by *t*-test.

To validate the accuracy of the RNA-Seq data, we selected 9 out of the 41 DEGs from the plant hormone signal transduction term for real-time RT-PCR analysis. When compared to the empty vector pTY-S-inoculated leaves, we found that 7 (*Bra011332*, *Bra014411*, *Bra023958*, *Bra027232*, *Bra005136*, *Bra017136*, and *Bra025708*) out of the 9 DEGs were significantly increased while the other 2 DEGs (*Bra003044* and *Bra022663*) were significantly decreased in the *BrAN3*-silencing plants (Figure 7). We also found the same expression patterns of these 9 DEGs were observed in the RNA-Seq data when calculated as the FPKM (Figure 7). Thus, the real-time RT-PCR and RNA-Seq data correlated very closely (Figure 7).

## Discussion

Leafy head formation in Chinese cabbage is a tightly controlled process. Many proteins such as the SWI/SNF chromatin remodeling protein complex, ATPases, and transcription activators could play important roles in head formation. This is the first time to functionally confirm that the *BrAN3* gene could induce head formation in Chinese cabbage. Its distinct expression patterns in different leaf locations and at different head developmental stages are consistent with its function in head formation. Similar expression patterns had also been observed for the *BrTCP* gene (Mao et al., 2014) and the *BrLAX*, *BrPIN*, *BrPGP* genes (Gao et al., 2017), which exhibited higher expression levels in the leaf apical tip and at the rosette stage than in the leaf base and at the heading stage in Chinese cabbage. However, the *BcpLH* (Yu et al., 2000), *BrGRF* (Wang et al., 2014a), and *BrpSPL9* (Wang et al., 2014b) genes showed a gradual increase in transcript abundance from the rosette stage to the heading stage, implying different functions.

Gene knockout/knockdown methods such as the CRISPR-Cas9 system, RNA interference, T-DNA insertion, transposons, and some chemical reagent-induced mutations suffer from the limitations of off-target effects, functional redundancy, embryonic lethality, and multi-insertions (Pflieger et al., 2008). To the contrary, VIGS is a rapid and cost-effective RNA-mediated reverse genetics technology, and does not rely on the acquisition of mutants or transgenic plants. It can be easily applied in many species to study gene function either individually or on a large scale. pTY-S is a newly developed VIGS vector, which contains the entire genome of TYMV, and the 35S CAMV promoter and terminator (Pflieger et al., 2008; Huang et al., 2018a,b; Muntha et al., 2018). It possesses a strong infection ability, produces liable and robust VIGS effects that lasts throughout the plant life (Pflieger et al., 2008), and has been successfully applied in Chinese cabbage (Yu et al., 2018). In the present study, the expression of *BrAN3* and *BrBRM* genes had been effectively knocked down by each silencing vector, and the formation of leafy heads had been observed in the inoculated Chinese cabbage plants at the early stage (Figure 3A). The results demonstrated the function of the *BrAN3* and *BrBRM* genes in leafy head formation in Chinese cabbage. Since the efficiency of VIGS is highly dependent on the virus-host affinity, and the infected TYMV eventually took over and killed the host plants at the later stage of head formation, it should be noted that the effect of knockdown of the *BrAN3* and *BrBRM* genes on leafy head formation could be better examined in stable transgenic China cabbage in the near future.

Hormones such as auxin and gibberellins play important roles in the adaxial-abaxial patterning during leaf development in Chinese cabbage (He et al., 2000; Yu et al., 2000; Wang et al., 2014a; Gao et al., 2017). The identification of 41 DEGs from the plant hormone signal transduction term in the *BrAN3*-silencing leaf by RNA-Seq further demonstrated that plant hormones are key players in leafy head formation. In the present study, we found that cytokinin plays an important role in leafy head formation in Chinese cabbage. The significant increase and decrease in the expression of the *BrARR4* and *BrCRF2* genes in the *BrAN3*-silencing plants (Figure 3B) indicate that *BrAN3* is involved in the cytokinin signaling pathway since both *BrARR4* and *BrCRF2* genes are cytokinin response regulators (Rashotte et al., 2006; Werner and Schmuelling, 2009; Holst et al., 2011; Vercruyssen et al., 2014).

**FIGURE 8:**
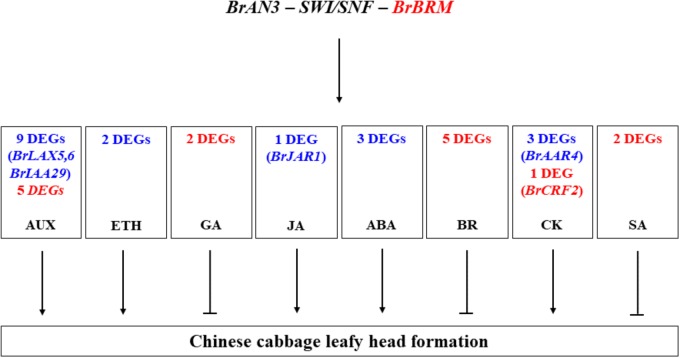
The potential roles of the signaling pathways of auxin (AUX), ethylene (ETH), gibberellin (GA), jasmonic acid (JA), abscisic acid (ABA), brassinosteroid (BR), cytokinine (CK), and salicylic acid (SA) in leafy head formation in Chinese cabbage. Red and blue font colors indicate the significantly induced and decreased DEGs detected by RNA-Seq in the present study. The genes in the parenthesis, which were functionally studied in previous studies, had been identified as the DEGs in the present study. Arrows represent the positive effects while inhibition arrows represents the negative effects.

Moreover, our RNA-Seq results revealed that the BrAN3 protein functions to induce the GA, BR, and SA signaling pathways, which inhibit leafy head formation in Chinese cabbage (Figure 6). The BrAN3 also inhibit the ethylene and JA signaling pathways, which promote leafy head formation in Chinese cabbage (Figure 6). The BrAN3 protein increases and decreases DEGs involved in the auxin, ABA, and CK signaling pathways, indicating the three pathways play both a positive and a negative roles in leafy head formation in Chinese cabbage (Figure 6). The accumulation and uneven distribution of auxin in the head leaves play an important role in Chinese cabbage leafy head formation (He et al., 2000; Gao et al., 2017). This is in accordance with our findings that a total of 20 auxin DEGs were significantly expressed in the *BrAN3*-silencing plants, including 13 up-regulated and 6 down-regulated DEGs. Among them, 3 DEGs (*Bra017136*, *Bra005136*, and *Bra 023958*, which correspond to *BrLAX5*, *BrLAX6*, and *BrIAA29*) had been demonstrated to be involved in leafy head formation in Chinese cabbage (Wang et al., 2012; Gao et al., 2017). Auxin mediates plant growth by affecting DELLA proteins (Fu and Harberd, 2003), while SWI3C, a core component of Arabidopsis SWI/SNF chromatin-remodeling complexes, also interacts with several DELLA proteins (Sarnowska et al., 2013). DELLA was reported as a repressor of ethylene signaling (Sarnowska et al., 2013), and interacts with JAZ in response of JA signaling (Lor and Olszewski, 2015; Vleesschauwer et al., 2016). Ethylene has been shown to induce petiole unequal growth between the adaxial and abaxial axes in Arabidopsis (Polko et al., 2012), and JAZ represses the JA signaling (Lor and Olszewski, 2015; Vleesschauwer et al., 2016). Accordingly, we found that the ethylene and JA signaling pathways were significantly induced in the *BrAN3*-silencing plants. The only DEG (*Bra000421*) which corresponds to the *BrJAR1* gene was identified to be a DEG in leafy head formation in Chinese cabbage by transcriptomics and proteomics analyses (Zhang C.W. et al., 2016). In addition, GA signaling was proved to be a positive regulator of the SWI/SNF chromatin remodeling complexes (Rafal et al., 2013; Jose, 2014). BRM regulates primary root elongation by repressing the activity of the ABA signaling pathway (Han et al., 2012). Our findings that the *BrAN3*-silencing plants inhibited and promoted GA and ABA genes expression, respectively, are consistent with those reports (Han et al., 2012; Rafal et al., 2013; Jose, 2014). Moreover, BRM binds to the promoter of *ARR16* for boundary establishment in Arabidopsis cotyledon, which is an inhibitor of CK responses (Efroni et al., 2013). Similarly, we found that 4 and 1 DEGs involved in the CK signaling pathway were up-regulated and down-regulated in the *BrAN3*-silencing plants, respectively (Figure 6). The potential roles of these hormone signaling pathways in leafy head formation in Chinese cabbage were summarized in Figure 8. Further studies should be conducted to investigate the involvement of each hormone signaling pathway in leafy head formation in Chinese cabbage.

## Conclusion

We identified and characterized the function of the *BrAN3* gene in leafy head formation in Chinese cabbage. We also found that DEGs involved in auxin, ethylene, GA, JA, ABA, BR, CK, and SA signaling pathways play either positive or negative roles in leafy head formation in Chinese cabbage. This information could be useful for genetic engineering, gene editing, and molecular breeding of Chinese cabbage to improve heading and thus yield.

## Author Contributions

CZ and XH conceived the project. JY and LG conducted the experiments. JY, LG, WL, CZ, DX, XH, JY, and TL performed the data analysis. JY, LG, WL, CZ, XH, and LS wrote the manuscript. All authors read and approved the manuscript.

## Conflict of Interest Statement

The authors declare that the research was conducted in the absence of any commercial or financial relationships that could be construed as a potential conflict of interest.

## Footnotes

1 http://brassicadb.org/brad/

2 https://phytozome.jgi.doe.gov/pz/portal.html#!search?show=BLAST

3 http://www.megasoftware.net/

4 http://brassicadb.org/brad/

5 http://bonsai.hgc.jp/mdehoon/software/cluster/

6 http://jtreeview.sourceforge.net/

7 www.omicshare.com/tools
