# Supplementary material for: Transcription Coactivator ANGUSTIFOLIA3 (AN3) Regulates Leafy Head Formation in Chinese Cabbage
Source: Front Plant Sci. 2019 Apr 30;10:520. doi: 10.3389/fpls.2019.00520 (PMC6502973; doi:10.3389/fpls.2019.00520)
Supplement: Supplementary file 2 [file Image_1.pdf]

Figure S1. Comparison of the homologs of the AtBRM protein in *Brassica rapa*, *B. oleracea*, *Zea mays*, and *Oryza sativa*.

(A) Phylogenetic relationship and gene structures of the homologs of the AtBRM protein in those species. The unrooted phylogenetic tree was generated by the neighbor-joining method using the MEGA5 program with 1,000 bootstraps. Lines represent the introns while solid boxes represent exons. (B) Comparison of the deduced amino acid sequences of the homologs of the AtBRM protein in those species.

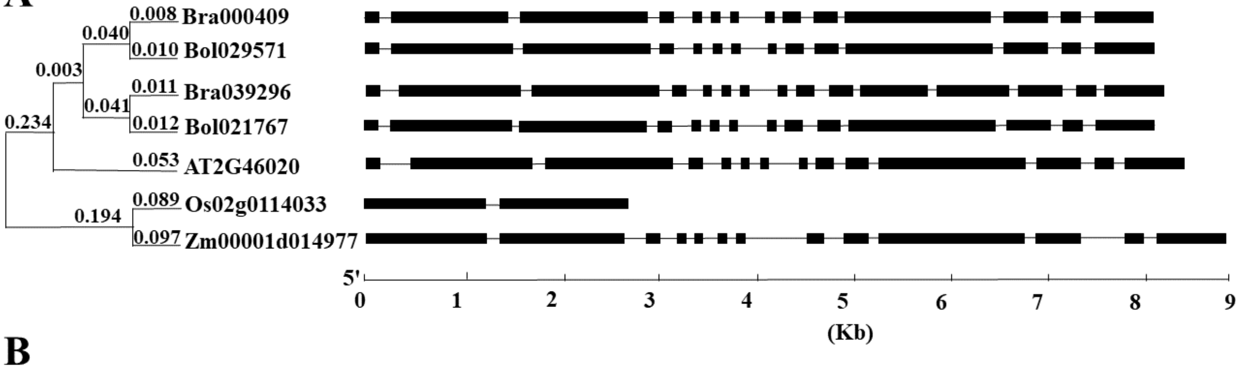

B

```
Bra000409         MQSGGSGGGPARNPGMGPGGRTNSTSSAASPSSSSSSVQQQQQQ----------LASRQQ    50
Bol029571         ............................................Q---------......    51
Bra039296         ..........................................HLA----------SRQ..    50
Bol021767         .L..........--....A............T............Q----------LASR.    48
AT2G46020         ..............A...A...A.....................QQQQQQQQQLASRQ..    60
Os02g0114033      -------------MQP.EAPPSSGG.P.T..RPPPAVA.G...LG----------FRNQA    37
Zm00001d014977    ------------------------------------------------------------     0

Bra000409         HQLRNSEGNDGMFAYHAGGAQGMMGAGSFG---SPSASMQ----PRKFFDSPQLQQ----    99
Bol029571         .........................G....---.......LPQQ............----   104
Bra039296         Q.G...A.........P..V.....G.N..---.S.G...QPQQ..RL.....Q..----   103
Bol021767         Q.G...A.........P..V.....G.N..---...G...HPQQ..RL.....Q..QQPQ   105
AT2G46020         Q.H...DT.EN....QP..V.....G.NFA---.SPG...MPQQS.N..E...Q..QQQQ   117
Os02g0114033      MMHHHDQQQQQQQG.PS.APH....G.GSSSFPPS.GP.PPFQGQ.NMPLP-GGP.G---    93
Zm00001d014977    MMHHHEQQQ----.F.S.APH....P.GVN-FPQS.GPVSSFQGQ.NLPL.SGGP.G---    52
                     ::.: :    .: .*. :**** *.      ....:      *.:  .    *

Bra000409         ---QHGEGQQSINPMQQAYIQFALQAQQQK----------AQQQARMGIMMGSSKDQDAR   146
Bol029571         ---...........................----------.H..................   151
Bra039296         --GSSQ.....F.......L..........----------........M..-........   150
Bol021767         --GSSQD....F.......L..........----------V..H....M.----------   143
AT2G46020         QGSSTQ....NF...........M...H..----------........MVG-..SVGKDQ   156
Os02g0114033      ---LA.GQ.HNPTA.....L.YMM.Q....---AHGMLLQQ....K.NMAGP.TR...VA   147
Zm00001d014977    ---MV.GQMHNQVA...QFLKL.M.Q...QQKAAQGMLLQQ--..K.NMAGS..R...ML   107

Bra000409         MGVLNMQEMMPMQASNMAQASSSRPAG------EQFSHGERQMESGPQQRNETKPHP-QQ   199
Bol029571         .............T...........S.------..LA......G.............-..   204
Bra039296         ..M....GA.......Q..G...N.SA------...AR........----H......-..   199
Bol021767         ------.GA.-Q....Q..G...N.SA------...AR........----H......-..   185
AT2G46020         DARMG.LN.QDLNP.SQP.....KPSG------D..AR....T..SS.......S..Q..   210
Os02g0114033      ANTAK...L.SL..QAQ..MFKRQQSEHLQQAEK.AEQ.QPSNSEQRSGDMRPPSM.P.G   207
Zm00001d014977    NNPAK...L.ALH---Q..MYKRQCE--------.KEQ.QSSGSEQRSGDMRPP-M.P.G   155
                              :    .*  . .          *  :*: .  .      ..   * *

Bra000409         VGTGQLMPGNIIRPMQAPQAMQGVNNMGTNQLALSQQWQAMQAWARERNIDLSHPANANQ   259
Bol029571         A...........K...............S....H........T.................   264
Bra039296         ...................GP............F........................S.   259
Bol021767         ...........T.......GP.......S....F........................S.   245
AT2G46020         ..........M.........Q.L.....N....FA.......................S.   270
Os02g0114033      .PGQ..SSAGMV....PM.GQA.MS.A.A.PM.M-A.L..I....K.H.V...N...VTL   266
Zm00001d014977    ..GQ..PSMGM.....PI.GQV.MGSA.G.PITP-T.F..I....K.H.F...N...ISA   214
                  .   ** . .: :***. *.   :.. * * ::   * **:*:**:*:*.***:*** .

Bra000409         MAHIL----QGRMAAQQKAIEGNVASQSPTMPVSSQPVSSSALPGENSPRANSASDISGQ   315
Bol029571         .S...----.A..................A............V.............F...   320
Bra039296         .S...----....V.....N.........SI......S...GV.......P...G.....   315
Bol021767         .S...----.........SN......P..SI......S...GV.......P...G.....   301
AT2G46020         .....----.A........G.........SI.I....A...VV......H..........   326
Os02g0114033      ISQ..PMLQSN....M..QN.VGM...QQ-SVP.QMN---NDA..HSNFPSQG.PSKPR.   322
Zm00001d014977    ISQL.PIWQSN....M..QN.ANM.A.QQQALP.QVN---.DT..HVNAPSQG.LLKPR.   271
                  ::::*    . **.* **  * .:*: .     *.     .  **. .  .:.*   . *
```

Fig. S1 (Cont.)

```
Bra000409          SGGPAKARHAN---SFASTSSPRMVNPAASPFS-QGRDNPMYPRHLVQPTNGMQSGNSLQ   371
Bol029571          ...........---...................-.............A.....V.......   376
Bra039296          ..-SG.....IPTS..........M....N...A......P.............P....M.   374
Bol021767          ..-SG.....ISTS..........M....N...A...................P....M.   360
AT2G46020          ..-S......LSTG..............MN...G...E...............P...P..   385
Os02g0114033       PLP.STSVSGGAEPKMMNM.N--.QMQQQLAAHNRDSS.DRAA.PAMSMG..G.MMHMP.   380
Zm00001d014977     PLA.S-SISGGEEAKVVNS.N--LQLQQQFPVHNRDGS.ERAV.S.MTGG..A.TTHIP.   328
                   .  .. :  .    .. . *.  :      .   :. .*    *      **    :  *

Bra000409          TSANDAHVLDQ----KKSLGSSEHLQMQQPRQLNAPTPNLAAPSDAGPLSNSSRQSGQGT   427
Bol029571          ....E.....----..R...........................................   432
Bra039296          ....ET....HNASTN.G...A..........M...S.-K.VI....L.N...L.....I   433
Bol021767          ....ET....HNASTN.G...A..........M...S.-K.VL....L..K..L.I...I   419
AT2G46020          ....ETP....NAST.....PA...........NT.....V....T.......L......   445
Os02g0114033       S.GHANKIPE.-PNP.N--AN..AM...YA...QQANRAT.PSANS.ETGG.--.APNQA   435
Zm00001d014977     S.GHVNKIPE.-PNP.NV.AN..AM...HV..MQQLNQAA.PT.TPIEAGG.--.VPTSA   385
                   :*.:   : ::    ::  . :* :***  **::  .   .  : .   . *  *

Bra000409          QQAQ-RPGFTKQQLHVLKAQILAFRRLKKGEGSLPQELLKAIAP-PPLELQTQRQVS-PV   484
Bol029571          ....Q.S.....................................-............-..   490
Bra039296          K.E.Q.S..........................F.....-....V..PL.IF-..   491
Bol021767          K.E.Q.S..........................F.S...-....V..PL.IF-..   477
AT2G46020          ....Q.S..........................P...Q..S.-...........I.-.A   503
Os02g0114033       ARPP--M....H................R.DKK..P.V.DL.MSG..PDS.-AQ...G.P   492
Zm00001d014977     .PQTGQT....N................R.DR-..P.V.EL.VSGR.PDS.G.Q...G.Q   444
                          ****:****************:*:  ** *::. * .  * : *   *:  *

Bra000409          RLQVQDRSTDKTVEDQARSLESG-KESQAAASSNGQIFSKEEENLRDTEVPLAKS--HSQ   541
Bol029571          ..................L....-..............................--...   547
Bra039296          .VHG....S.....N........-....T.............D.VGG......TG--...   548
Bol021767          KVH.....S.....N......P.-....T.........A...D.GG.......M.--...   534
AT2G46020          IGK.....S...G........C.-...........P......D.VG....A.TTG--...   560
Os02g0114033       VTN-RE..ATSSADEHG.PV...GIAPERSSLLKAPCLP.V.VSAPEDKTIPASG--PM.   549
Zm00001d014977     VTHNRE.P.VSNADEHGKQM...G.APEKP.LLK.PCLP.V.VSTSEDKASP.SGPGPA.   504
                     : ::*.: .. :::.: :* *   .: .:  :.  :.* * .    :.  : .    *

Bra000409          LFQNRGKEPASIDAVTKLEQQTDVLPVKSDQGAEASTQQTP-RSDSNADKGKAVASDGDQ   600
Bol029571          .......G................F................-..................   606
Bra039296          ....L...A..TA.A..E...A..F........D.......-................G.   607
Bol021767          ....L...A..TT.A..E......F........D.......-................G.   593
AT2G46020          ....L...AT.T.VA..E......F........DS...KN.-....T...........S.   619
Os02g0114033       VMKASP...LR.GP.S-MPE..NTTLI..E.DP.RGI.R..G...Y.GER..SLPAESGS   608
Zm00001d014977     VMKASP...LK.GP.S-VPEHCNTTVI..E.DL.RNI.R..G...Y..ER..SVPAESGS   563
                   :::   * .     .:   :: :.  :**:*. : . *:.* *** ..::**::.::...

Bra000409          SNVPSQANTPQQPKDTAS----ARKYHGPLFDFPFFTRKHDSYGSATANANNLTLAYDI   656
Bol029571          .............H.....----......................................   662
Bra039296          ....P...S........P----..................T...............   663
Bol021767          ....A...S.........----................Y.T...............   649
AT2G46020          .K..P...S..P......----....Y...........L..................   675
Os02g0114033       ADAEQAKRAASSSSVPTPNRDVS...........S.......MV..NY.S.--.A.G..V   666
Zm00001d014977     VD.EQAKR.GSTSSAPVP-RDVP...........S...R...M...NY.S.--.S.G..V   620
                    ..    .: .  . ...    .***:******* ***: *:  **. *:*  *:*.**:

Bra000409          KDLICEEGAEFFNKKRANSLKKINGLLAKNLERKRIRPDLVLRLQIEEKKLRLRDLQSRV   716
Bol029571          .................D..........................................   722
Bra039296          ...V..........TD..N.......T.........................SA.....   723
Bol021767          ..............TD..T.......R.........................SA.....   709
AT2G46020          ...........LS...TD...................................S......   735
Os02g0114033       ....AQ..MIVLG...EDN....S....I.......Q..............K.LEF.A.M   726
Zm00001d014977     ...LAQ..MIVLGR..EDN....S....I.......................LEH.A.L   680
                   ***:.:**  .:.:** :.*.**.**** *******:**************:*   *:*:
```

Fig. S1 (Cont.)

```
Bra000409           RDEVDRQQQEIMSMPDRPYRKFVRLCERQRLEMNRQVLASQKAVREKQLKTIFQWRKKLL   776
Bol029571           ............................................................   782
Bra039296           .........D.............................N....................   783
Bol021767           .........D.............................N....................   769
AT2G46020           .E.....................................N....................   795
Os02g0114033        ....EQE.....A....V......Q.....V.LT...QQM...S......S.........   786
Zm00001d014977      ....EHE.....A....I......Q.....V.LV...QQM.R.S......S.........   740
                    *:**:::**:**:**** ****** *****:*: ***   *:* ******:*********

Bra000409           EAHWAIRDARTARNRGVAKYHEKMLREFSKRKDDGRNKRMEALKNNDVERYREMLLEQQT   836
Bol029571           ............................................................   842
Bra039296           ...............................P............................   843
Bol021767           ....S..........................P............................   829
AT2G46020           ............................................................   855
Os02g0114033        ..........IT..........R.......K...D.................QI......   846
Zm00001d014977      ..........IT..........R.......K...D.................QI......   800
                    ****:***** :**********:*******: **.*****************::******

Bra000409           NIPGDAAERYAVLSSFLTQTEDYLHKLGGKITATKNQQEVEEAANAAAVAARLQGLSEEE   896
Bol029571           ................................D...........................   902
Bra039296           ..........N.....................................I...........   903
Bol021767           ..........N.....................................I...........   889
AT2G46020           .M..........................................................   915
Os02g0114033        SV.....Q..N..........E..Y........A..H.Q.........A...A.......   906
Zm00001d014977      SV.....Q..N..........E..Y..........S..Q.........A...A.......   860
                    .:*****:** **********:**:******* :*.:*:********* *** *******

Bra000409           VRAAAACAREEVVIRNRFMEMNAPKDNSSVNKYYTLAHAVNELVVRQPSMLQAGTLRDYQ   956
Bol029571           ............................................................   962
Bra039296           ............L.....V.......................V.................   963
Bol021767           ............L.....V.......................V.................   949
AT2G46020           .....T............T......E................V.................   975
Os02g0114033        .K...Q..GQ..M...T.S.....RE.T..............R.T....L.R........   966
Zm00001d014977      .K...Q..GQ..M...T.S.....R..T............S.R.TK...L.R........   920
                    *:*** ** :**:*** * *****::*:************.* *.:***:*:********

Bra000409           LVGLQWMLSLYNNKLNGILADEMGLGKTVQVMALIAYLMEFKGNYGPHLIIVPNAVLVNW  1016
Bol029571           ............................................................  1022
Bra039296           ............................................................  1023
Bol021767           ............................................................  1009
AT2G46020           ............................................................  1035
Os02g0114033        ................................S...........................  1026
Zm00001d014977      ..................................V.........................   980
                    ********************************:*:*************************

Bra000409           KSELHTWLPSVSCIYYVGTKDQRSKLFAQEVCAMKFNVLVTTYEFIMYDRSKLSKVDWKY  1076
Bol029571           ............................................................  1082
Bra039296           ...........................S................................  1083
Bol021767           ...........................S.....L..........................  1069
AT2G46020           ...........................S-Q.KFE..........................  1094
Os02g0114033        .----------------------------..L.V...........V........RI....  1058
Zm00001d014977      ....LN....A...F...A....Q...S...M.............V.F......R.....  1040
                    *                            :*   ***********:*:******::****

Bra000409           IIIDEAQRMKDRESVLARDLDRYRCQRRLLLTGTPLQNDLKELWSLLNLLLPDVFDNRKA  1136
Bol029571           ............................................................  1142
Bra039296           .V..........................................................  1143
Bol021767           .V..........................................................  1129
AT2G46020           ............................................................  1154
Os02g0114033        ....................................................E.......  1118
Zm00001d014977      ............D.......................................E...SS..  1100
                    *:**********:***************************************:***. **
```

Fig. S1 (Cont.)

```
Bra000409           FHDWFAQPFQREGPAHN-IEDDWLETEKKVIVIHRLHQILEPFMLRRRVEDVEGSLPPK-  1194
Bol029571           .................-.........................................-  1200
Bra039296           ..............S..-.........................................-  1201
Bol021767           ..............S..-.........................................-  1187
AT2G46020           .................-.......................................A.-  1212
Os02g0114033        .Q...SK....DV.T.SEE............I.........................R.V  1178
Zm00001d014977      .S...SK....D..T..EE............I.........................R.-  1159
                    * ***::***:: *:*.  ************:************************* *

Bra000409           ------------------------------VSVVLRCRMSAIQSAVYDWIKATGTLRVDP  1224
Bol029571           ------------------------------..............................  1230
Bra039296           ------------------------------..........S...................  1231
Bol021767           ------------------------------..........S...................  1217
AT2G46020           ------------------------------..............................  1242
Os02g0114033        DCSALVTLSDAICHSALFVCYIVNSSCHLQE.I.......G..G.I.....S...I....  1238
Zm00001d014977      ------------------------------D.I........V.G.I.....S...I....  1189
                                                   *:*******.:*.*:*****:***:****

Bra000409           DDEKLRAQKSSIYQAKIYRTLNNRCMELRKTCNHPLLNYPYFNDLSKDFLVRSCGKLWIL  1284
Bol029571           ......T.....................................................  1290
Bra039296           .....K...NP.......K.........................................  1291
Bol021767           .....K...NP.......K.........................................  1277
AT2G46020           .........NP...................A.............F...............  1302
Os02g0114033        E...A.I.RNAM....T.KN...K......V......S..FM.YYG...II.......N.  1298
Zm00001d014977      E...R...RNPM..V.T.KN...K......V......S..FL.-HG...MI.......N.  1248
                    :*** : *:..:**.* *:.***:******.******.**::*  .***::******* *

Bra000409           DRILIKLQRTGHRVLLFSTMTKLLDILEEYLQWRRLVYRRIDGTTSLEDRESAIVDFNDP  1344
Bol029571           ............................................................  1350
Bra039296           ............................................................  1351
Bol021767           ............................................................  1337
AT2G46020           ............................................................  1362
Os02g0114033        .......H.S........................Q.......................R.  1358
Zm00001d014977      .......HKS................M.D.............................R.  1308
                    *******:::****************:*:*****:*********************** *

Bra000409           DTDCFIFLLSIRAAGRGLNLQTADTVVIYDPDPNPKNEEQAVARAHRIGQTREVKVIYME  1404
Bol029571           ............................................................  1410
Bra039296           ............................................................  1411
Bol021767           ............................................................  1397
AT2G46020           ............................................................  1422
Os02g0114033        NS...................S.............Q................D.......  1418
Zm00001d014977      GS...................S.............Q........................  1368
                    .:*******************:*************:****************:*******

Bra000409           AVVEKFSSHQKEDELRSGGSIDVEDDLAGKDRYIGSIEGLIRNNIQQYKIDMADEVINAG  1464
Bol029571           ............................................................  1470
Bra039296           .....I..............M.L........------------------------L....  1447
Bol021767           .....M..............V.L.....................................  1457
AT2G46020           .....L..............V.L...M.................................  1482
Os02g0114033        ...DNI..Y.......N...G.L..........M....S.....................  1478
Zm00001d014977      ...DNI..Y.......N...G.L..........M..........................  1428
                    ***:::**:*******.*** *:***:****                        :****

Bra000409           RFDQRTTHEERRMTLETLLHDEERYQETVHDVPSLHEVNRMIARSEEEVELFDQMDEEFD  1524
Bol029571           ............................................................  1530
Bra039296           .....................D......................................  1507
Bol021767           ............................................................  1517
AT2G46020           ............................................................  1542
Os02g0114033        ...................................QQ.......T...............  1538
Zm00001d014977      ..........................DS.......Q........T.S..........D..  1488
                    *********************:****::*******::*******:*.**********:**
```

Fig. S1 (Cont.)

```
Bra000409          WTEEMTSHEQVPKWLRASTREVNTTVADLSKKPSKNMLSSSNLIVQTAGPGGERKRGRPK  1584
Bol029571          ............................................................  1590
Bra039296          ......C........................................G............  1567
Bol021767          ......C................A.......................G............  1577
AT2G46020          ......N................A......................PG............  1602
Os02g0114033       ..GD.MK.N.A.....V.ST.LDAV..S.....LR..AAGG--.SLDTNEKL.KR.....  1596
Zm00001d014977     ..GD..K.H.......VNSN..DAV..S......R..S.GG--.ALDTNETL.KR....R  1546
                   ** :*  *.*.*****..: *:::.**.***** :** :..  *    .   *::****:

Bra000409          S--KKINYKEIEDDIGGYSEESSDERNIDSGNEEEGDIEQFDDDELTGALGNHQTNKDES  1642
Bol029571          .--....................E......................I.............  1648
Bra039296          .--.............LF.....E.V.V..........G.S..........DQ...NG..  1625
Bol021767          .--.............LF.....E.V.V............S..........DQ...NG..  1635
AT2G46020          .--............A.......E..............R............D.....G.F  1660
Os02g0114033       GSG.YSI.R..D..DFEE.DDD.E...TS.-LP...E.GE.E.E.DNDDS--VPD...Q.  1653
Zm00001d014977     GTG.YSI.R..D.EDLEE.D.D.E...TA.-LP...EVGE.E.E.DNDDS--VPD.....  1603
                   .  *   *:**:*:     *::.*:* *  *   ***:: : :*:*  .       *:.:

Bra000409          DGENPVRGYDYPQRSGCYKKNTPR-DDAGSSGSSPESHRSKEMASPVSSRKFGSLSALDT  1701
Bol029571          ....................I...-...................................  1707
Bra039296          ......A.....P...S...VP.Q-......E.................K..........  1684
Bol021767          ......A.....P...S...VP.Q-......E.................K..........  1694
AT2G46020          ......C.....PG..S....P..-........................Q..........  1719
Os02g0114033       EE.E.INDEG.DFSH.MGRRKAH.SEE...T...SGGR.LPPP.PSS..K.LR......S  1713
Zm00001d014977     EE.E.MNDDV.EFTE.LRGRKAN.MEE...T...SG.R.LPPPVPSS..K.LR......A  1663
                   : *:*: .   *     *    : . : ::***: **. .:*     ... **:*: ******:

Bra000409          RPGSVSKRLVDDTEDGEIAASGDSHIDLQRSGSWAHERDEG-EEQVLQPTIKRKRSIRLR  1760
Bol029571          .........................................-..................  1766
Bra039296          ............L.....G............----YD....G..................  1740
Bol021767          ............L.....G............----YD....GG.................  1750
AT2G46020          .........L..L.E.....................D....D..................  1779
Os02g0114033       ...AL...TA..L.E....L.....L...Q....N....D.ED...V..K........I.  1773
Zm00001d014977     ...TL...TP..L.E....M.....M...Q....N....D.ED......K..........  1723
                   ***::***  ** *:***. *****:***:*     ::**:*  ***:**.********:*

Bra000409          PRQTGERIDGTDMPAAQP-------LQVNPSYRSKLRTVVDSHGSRQEQSDSSSRLRSLP  1813
Bol029571          ..................-------...DR..............................  1819
Bra039296          ....A.G..VS.V.....-------...DR.........A.......D............  1793
Bol021767          ....A.GT..S.......-------..GDR.........A.......D............  1803
AT2G46020          ....A..V..SE......-------...DR.............S...D...........V.  1832
Os02g0114033       ..PNA.KL.DRSGDGTV.QRGVHLAF.GDGD.D.QFKSEQ-AFADPASRQQD--TVHRTV  1830
Zm00001d014977     .KPNA.KQEDRSGEG.F.QRGTHLAF.GDGH.D.QFKSDL.ARAFPAARQQD--AVHPIV  1781
                   *: ..*  :  .  .: *        :* :  * *::::   : .    :.:.   ::

Bra000409          GKKIANTSKLHVSSPKSGRLNTTQLPLEDNTEAARETWDG--TSPIGSSNAGARMSHNIQ  1871
Bol029571          ...V....................................--..................  1877
Bra039296          A..V.................A....V...D.........--...............T..  1851
Bol021767          A..V...........T.....A....V...D.........--..H............I..  1861
AT2G46020          A..V.S...............A...TV...A..S......--....S..........I..  1890
Os02g0114033       KQ.RNMP.RKAPPAT.A.KMTQLSGSG.GSA.HSK.N.SNKVIESA.PNSS.TK..DSM.  1890
Zm00001d014977     KQ.RNMS.RKVSPASR..K.SHLSGSG.GSA.LSK.N.SSKAIDSTAPEFG.TK..DSM.  1841
                    :*   .*:   .:. :*::.  . . *.. * ::*.*..    .   ... .*::**. :*

Bra000409          KRCKTVISKLQRRIDKEGQQIVPMLTNLWKRIQTGYAAGGVNNLLELREIDHRLERLEYA  1931
Bol029571          ............................................................  1937
Bra039296          .................................N.................Q.V.....V  1911
Bol021767          .................................N.................Q.V.....L  1921
AT2G46020          ....I............................N...................V......  1950
Os02g0114033       RK..N..N..W.......H..I.NISSW.R.NENSSFKGLASST.D.QK.EQ.VDGF..G  1950
Zm00001d014977     RK..N.....W.......H.MI.NISSW.R.NENSSFR.PAGST.D.QK.EQ.VDG...G  1901
                   ::** **.** *******:*::* ::. *:* :..    * ... *:*::*::*:: :**
```

Fig. S1 (Cont.)

```
Bra000409        GVMELASDVQYMLRGAMQFYGFSHEVRSEARKVHNLFFDLLKMSFPDSDFREARNALSFS  1991
Bol029571        ..................................................G.........  1997
Bra039296        ...............................................T............  1971
Bol021767        ...............................................T............  1981
AT2G46020        ..........L...................K................T............  2010
Os02g0114033     ..N.FIA.M.Q..KSVV.HFSYR....V..ETL.....NIM.IA.........KG.M...  2010
Zm00001d014977   A.T.FIA.M.Q..KSVV.HFSYR....I..ETL.....NIM.IA......M..K..M...  1961
                 .* *: :*:* **:..:*.:.: **** **..:*****::*::***:**  **:.*:***

Bra000409        GPTPTLVSTLSPRTVG-ISQGKKQK----PVNEEEPE-PSSPQRPQQREN------SRIR  2039
Bol029571        .............A..-.N......----........-............------....  2045
Bra039296        ..S......S...GAVG.....RP.----..D.....E......R.....------....  2021
Bol021767        ..S......S...GA.G.....RP.----..D.....-...----.....------....  2026
AT2G46020        .SA......PT..GA.-.....R..----L...P.T.-......S.....------....  2058
Os02g0114033     N.GGGASGSAAQS.KQSA.GQ.RRS----STS.A.QHGS.--TSRHNQHAPVGEVSG.AH  2064
Zm00001d014977   N.GSGAAAAP.SSKHAAP.LKRSRASASASAS.A.QQHG.GHSSRHNQPSEAVPPS-.SH  2020
                 ..     .: :       .  :        ..* * .  *     :::         * :

Bra000409        VQIPQKETKLGGTSS---HNDDSPILAHPGELVICKKKRKD--REKSAPRTRTAGSSSPV  2094
Bol029571        ...............---.......................--.................  2100
Bra039296        ......DP.......---.T.E...................--.................  2076
Bol021767        ...............---.T.E...................--....G............  2081
AT2G46020        .............T.---.T.E...................--....G.K...G......  2113
Os02g0114033     TSKSE.DSRHS.PG.REQFT.SAGLFR..TDMF.V....DR--.PSLGSPSSSGR-TG.L  2121
Zm00001d014977   SSRSERDPRH..SRD-QHLQ.GAAG.L..SDMF.V....QERA.SGIGSPSSSGRGAG.L  2079
                  . .:::.: .*. .     * :  : ** ::.* ****.   *   .. : :.  :.*:

Bra000409        SP-------PAMVGRGLRSPVSGSG----TRETRLAQQQRWPNQGTHPNNSGAAGDS---  2140
Bol029571        ..-------................----............................---  2146
Bra039296        ..-------Q..I...........V----........-------A............---  2115
Bol021767        ..-------Q..I...R.......V----........-------A............---  2120
AT2G46020        ..P------...I..........GV----P..............P............---  2160
Os02g0114033     ..TNAGRMG..PSP..A.T.FQRDP--HPSQQSMHSAG--.GAHSVQQSDR.GSSS--PG  2175
Zm00001d014977   ..ANPGRPG..PSP..A.T.FQRDPPPHPSQQSMHSAGSG.GSG.A.SDHQAG.SS.APG  2139
                 **        *   ** *:*.. .     .:::  :         .: .. ..:..

Bra000409        ---VGWANPVKRLRTDSGKRRPSHL  2162
Bol029571        ---......................  2168
Bra039296        ---......................  2137
Bol021767        ---......................  2142
AT2G46020        ---......................  2182
Os02g0114033     IGDIQ..K.T..S...........M  2200
Zm00001d014977   IGDIQ..K.A......RAG.GGRA-  2163
                    : **:*.** *** . *
```
